# The Effects of Division of the Vagi upon the Heart

**Published:** 1883-03

**Authors:** A. M. Bleile, A. Feil

**Affiliations:** Columbus, O.; Columbus, O.


### THE EFFECTS OF DIVISION OF THE VAGI UPON THE HEART.

BY A. M. BLEILE, M.D., AND A. FEIL, COLUMBUS, O.

The following work was carried out in the physiological laboratory of Starling College. Our object was the demonstration of trophic nerves. The existence of nerve fibres regulating nutrition only, or trophic fibres, has been, though still with some dispute, generally accepted in physiology. This acceptance rests on circumstantial rather than direct evidence. In searching after these fibres by cutting a nerve trunk supplying an organ and then noting the changes produced thereby in such organ, the problem is complex, because on the one hand sensory fibres are destroyed and thus sensation in the part is abolished, which sensation protects from prolonged injury by giving prompt notice and calling forth means for checking injurious action; on the other hand, the vaso-motor mechanism is interfered with, and it is not easy to say what part the altered state of the blood-vessels may play in the production of changes seen after nerve section. So, also, division of a nerve entails in many cases loss of function, which alone may cause changes without any nervous intervention. As a first object for our search the heart and its vagus nerves seemed to us the most suitable; for, first, the vagus has no vaso-motor fibres to the heart, and secondly, the heart goes on beating after division of one or both nerves, the beat being only changed in frequency, which change subsides to a greater or less degree in time. In fact after division of only one vagus the change is quite temporary, and in a very short time the normal status is nearly if not completely reached.

Mammals on which section of both vagi has been performed, as is well known, almost invariably die of pneumonia, about the causation of which there has been much discussion, but this is a point which does not further concern us here. Suffice it to say that it is probably caused by the penetration of saliva and food matter through the trachea and into the lungs from interference with swallowing. It can generally be prevented by tying in a tracheal canula, but the animals die, nevertheless.

Eichhorst found that pigeons did not suffer this lung change, yet died, and not of inanition, as he proved. He found a change which had hitherto been overlooked, namely, a fatty degeneration of the heart, and to this he ascribed the death of the animals. He also argued from this for the presence of trophic nerve fibres for the heart, as the change could not be otherwise explained. The factor of increased frequency of the heartbeats had nothing to do with the degeneration, for frequency brought about by the continued use of atropin entailed no change whatever in the structure of the heart.

As already stated, the vagus has no vaso-motor effects on the heart. He also experimented in a very few cases on rabbits and dogs, in all cases cutting both vagi, and arrived at similar conclusions.

Knoll, Zander and others gave as a cause of death and heart changes in animals in which both vagi had been cut and pneumonia prevented, inanition from inability to swallow food.

Our experiments differ from those cited in this important point. In nearly all cases one vagus was cut. In a few cases both vagi were cut. We give a few examples: Rabbit No. 1, divided both vagi; death in twenty-three hours. Rabbit No. 4, divided both vagi; death in thirty-six hours. In these cases both lungs were affected nearly throughout their whole extent. Rabbit No. 6, divided left vagus; rabbit to all intents and purposes normal; killed on fifth day; at autopsy pregnancy was revealed. Rabbit No. 7, divided right vagus; rabbit normal; killed on fifteenth day. Rabbit No. 9, divided left vagus; rabbit pregnant at the time. On seventh day had a litter of seven rabbits. Everything normal so far as could be observed; killed by accident on thirty-second day. Portions of each heart were examined fresh. The organ was then put in ¼ per cent. chromic acid; after two weeks transferred to 70 per cent. alcohol and kept in this until required for use.

Staining agents used were osmic acid, carmine and the most satisfactory Bismark brown, some of which we obtained from Prof. Tuttle.

In all the hearts and in both auricles and ventricles nearly all the fibres were somewhat swollen, many of them showed an uneven contour, the cross-striæ had disappeared and in their stead we found an exquisite granular degeneration, probably fatty, the granules in most cases being as regularly disposed as though struck with a die. Here and there were fibres still showing striæ in parts, while in other parts the granulations are well marked. Normal fibres were extremely rare. Leaving out those cases in which both vagi had been cut, the animals a few hours after the operation were to all intents in a normal state; nor was anything abnormal revealed by post-mortem examination: the lungs were intact in every case. Yet all the hearts presented plain evidences of degenerative change, which we think cannot be explained otherwise than by the assumption of trophic cardiac fibres in the vagi, and that division of one vagus only is sufficient to destroy the normal balance and bring on degeneration. Our results further differ from Eichhorst in that, judging from his plates, the degeneration has not advanced as far as in his cases. Whether a longer time would bring about the further change, or whether the trophic impulses by the other vagus are sufficient to prevent it, we are unable to say.—*Proceedings American Society Microscopists.*

---

Shaving the Quinine-Tree.—The Ceylon *Observer* reports the practice pursued for some years of shaving bark off living cinchona trees has not been productive of any ill effects. The bark "renews" with even a larger percentage of alkaloid. Some trees have been thus treated on five successive occasions without their vitality having been in the least impaired.

